# Conflict Tasks of Different Types Divergently Affect the Attentional Processing of Gaze and Arrow

**DOI:** 10.1177/2041669518771713

**Published:** 2018-05-07

**Authors:** Lingxia Fan, Huan Yu, Xuemin Zhang, Qing Feng, Mengdan Sun, Mengsi Xu

**Affiliations:** Beijing Key Laboratory of Applied Experimental Psychology, National Demonstration Center for Experimental Psychology Education, Faculty of Psychology, 47836 Beijing Normal University, Beijing, China; Beijing Key Laboratory of Applied Experimental Psychology, National Demonstration Center for Experimental Psychology Education, Faculty of Psychology, Beijing Normal University, Beijing, China; State Key Laboratory of Cognitive Neuroscience and Learning and IDG/McGovern Institute for Brain Research, Beijing Normal University, Beijing, China; Center for Collaboration and Innovation in Brain and Learning Sciences, Beijing Normal University, Beijing, China; Beijing Key Laboratory of Applied Experimental Psychology, National Demonstration Center for Experimental Psychology Education, Faculty of Psychology, Beijing Normal University, Beijing, China; School of Psychology, Southwest University, Chongqing, China

**Keywords:** gaze, arrow, attentional process, conflict

## Abstract

The present study explored the attentional processing mechanisms of gaze and arrow cues in two different types of conflict tasks. In Experiment 1, participants performed a flanker task in which gaze and arrow cues were presented as central targets or bilateral distractors. The congruency between the direction of the target and the distractors was manipulated. Results showed that arrow distractors greatly interfered with the attentional processing of gaze, while the processing of arrow direction was immune to conflict from gaze distractors. Using a spatial compatibility task, Experiment 2 explored the conflict effects exerted on gaze and arrow processing by their relative spatial locations. When the direction of the arrow was in conflict with its spatial layout on screen, response times were slowed; however, the encoding of gaze was unaffected by spatial location. In general, processing to an arrow cue is less influenced by bilateral gaze cues but is affected by irrelevant spatial information, while processing to a gaze cue is greatly disturbed by bilateral arrows but is unaffected by irrelevant spatial information. Different effects on gaze and arrow cues by different types of conflicts may reflect two relatively distinct specific modes of the attentional process.

## Introduction

Throughout our daily lives, our senses are bombarded with so many stimuli that our attentional system needs to select the most relevant information for further processing. To rapidly and effectively process relevant information, individuals have to use various directional cues to orient their attention to a related target rather than unrelated distractors. Two types of directional cues have been discussed frequently in previous studies: symbolic cues, such as pointing arrows or words with directional meaning, and social cues, such as gaze direction or pointing gestures (Nummenmaa & Hietanen, 2009; Ponkanen, Alhoniemi, Leppanen, & Hietanen, 2011). Several researchers have raised concerns about whether the attentional mechanisms adopted while attending to gaze and arrow cues are the same or different (Friesen, Halvorson, & Graham, 2011; Marotta, Lupianez, Martella, & Casagrande, 2012; Stevens, West, Al-Aidroos, Weger, & Pratt, 2008).

Some researchers assume that gaze may trigger a reflexive attentional orientation (Feng & Zhang, 2014; Frischen, Bayliss, & Tipper, 2007; Hadjikhani, Hoge, Snyder, & de Gelder, 2008; Li, Zhang, Song, & Feng, 2010; Zorzi, Mapelli, Rusconi, & Umiltà, 2003). There are at least two alternative standards regarding the reflexive attention of cues in the domain of attentional shifting. One such standard is that the rapid cue-induced facilitation effect can be found even when the cues are uninformative or task-irrelevant (Cheal & Lyon, 1991; Friesen & Kingstone, 1998; Jonides, 1981; Marotta, Casagrande, & Lupiáñez, 2013; Müller & Rabbitt, 1989). Thus facilitation, which results in faster responses for cued target, occurs even at short cue-target stimulus onset asynchronies, and even when the direction of the cue is irrelevant at indicating a subsequent target location (Langton & Bruce, 1999). Based on this standard, event-related potential studies have provided evidence that significant gaze-congruent early directing attention negativity and anterior directing attention negativity are observed after the appearance of gaze cues, indicating that reflexive attention shifts to the gaze-cued location occur in advance of target presentation even when the cue is unpredictable (Feng & Zhang, 2014; Li et al., 2010). A second important standard for reflexive attention is related to the resistance to cognitive capacity limitation. For example, some studies have recently shown that the gaze-induced orienting effect of a target is not modulated by dual-task load, indicating that the process of coding gaze cues is highly reflexive (Hayward & Ristic, 2013; Law, Langton, & Logie, 2010). Nevertheless, other studies have demonstrated that a secondary, resource-consuming task reduced the gaze-cuing effects, thus challenging the view that attentional orientations induced by gaze cues meet the reflexive criterion of resisting capacity limitation (Bobak & Langton, 2015; Pecchinenda & Petrucci, 2016).

Even so, studies exploring whether central arrow cues cause reflexive orienting similar to that caused by gaze cues provide more evidence to support the reflexive characteristic of both gaze and arrow cues (Bonato, Priftis, Marenzi, & Zorzi, 2009; Tipples, 2002; Tipples, 2008). For instance, when a gaze cue or arrow cue was presented before the target as uninformative distractors that were incongruent with the upcoming target location, equal interference effects on tasks were found following the presentation of the gaze and arrow cues, indicating that a gaze cue as well as an arrow induced reflexive attention and they could not to be ignored (Galfano et al., 2012; Kuhn & Kingstone, 2009; Stevens et al., 2008). However, while both arrow and gaze cues might trigger reflexive shifts in spatial attention, results from other studies revealed that a gaze cue might trigger stronger reflexive attention and it may be coded by a specialized mechanism (Ristic, Wright, & Kingstone, 2007; Zorzi et al., 2003). In their view, there might be a more stringent standard that was considered as a judgment of stronger reflexive attention. The standard pertained to whether the facilitation effect induced by counterpredictive cues could be strategically suppressed or modulated by factors such as cue-target colour contingencies or stimuli’s spatial coding (Driver et al., 1999; Pratt & Hommel, 2003; Ristic et al., 2007; Zorzi et al., 2003). Here, counterpredictive means that the cue appearing before the target is more likely to indicate the opposite position of the following target. In this regard, Friesen, Ristic, and Kingstone (2004) compared performances produced by gaze and arrow cues when each of these was counterpredictive. Specifically, if a participant saw a gaze cue or an arrow cue indicating the left location, the target was highly likely to appear at the opposite location. The results showed that gaze cues, rather than arrow cues, triggered a reflexive shift in attention to the cued location, implying that participants were unable to avoid attending reflexively to the location that the eyes were directing to, but they were able to avoid attending reflexively to the location indicated by the arrow. Consequently, they drew a conclusion that orienting to a gaze cue was more strongly reflexive because the gaze-induced reflexive attention was stable and relatively resistant to cognitive control. Thus, it is inconclusive whether gaze processing represents a unique attentional process in triggering reflexive orienting or if it shares attentional mechanisms that are similar to those used in arrow processing.

The previously mentioned studies primarily manipulated gaze and arrow cues separately in each trial. Few researchers have discussed the interactional effects when both such cues appear simultaneously and compete for the attentional resources. In a study conducted by Nummenmaa and Hietanen (2009), gaze and arrow cues were presented simultaneously, and participants were instructed to attend only to the gaze or arrow cue appearing before the laterally presented target in one trial, where the unattended directional cue served as a distractor. The conflict situation enabled the researchers to investigate how the attentional systems solve the potential conflict resulting from two centrally presented attentional cues. The results showed that gaze and arrow distractors resulted in similar attentional cuing effects, indicating that attentional orienting processes via gaze and arrow cues are equally reflexive. However, in their study, gaze and arrow cues were both presented inside a single schematic face, which may destroy the intact perception of gaze. The present study sought to further probe into how gaze and arrow stimuli are processed and whether they interact with each other when they appear simultaneously but indicate opposite locations, such as in a flanker task. The flanker task paradigm, also called the bilateral task paradigm, was originally developed by Eriksen and Eriksen (1974) and requires participants to respond to a target stimulus presented at fixation while ignoring flanker stimuli presented on either side of the target. This tests how effectively the target can be focused on and the distractors can be inhibited (Horstmann, Borgstedt, & Heumann, 2006; Stins, Polderman, Boomsma, & Geus, 2007). There are a few studies that have probed the processing of eye-gaze or arrows in flanker tasks, with previous findings suggesting that human faces may be a special class of visual stimuli for the human attentional systems (Dichter & Belger, 2007; Federico, Marotta, Adriani, Maccari, & Casagrande, 2013; Marotta & Casagrande, 2017). However, one limitation of the previous work is the lack of emphasis on how gaze and arrows direct attention when they appear simultaneously, thus competing for limited resources.

Experiment 1 of the present study directly explored the attentional processing mechanisms of gaze and arrow cues when they were presented as targets or distractors in a flanker task by comparing the facilitation or inhibition effects produced by each. Participants were instructed to respond to the direction of the central target (a gaze or arrow cue directing left or right) with two bilateral distractors (a gaze or arrow cue directing left or right). Building on the previous studies, the processing mechanisms of gaze and arrows may be different or the same. If gaze and arrow cues share a similar attentional processing pattern, equal flanker effects, as reflected by faster reaction times in congruent-direction trials than that in incongruent-direction trials, can be found when responding to a gaze target with bilateral arrow distractors and to an arrow target with bilateral gaze distractors. However, if the processing of a gaze cue triggers the reflexive orientation of attention more strongly than that of an arrow cue does, it will be less influenced by bilateral arrow distractors and would produce more interference in the processing of the arrow direction when presented as a bilateral distractor. In contrast, more reflexive arrow cue will be less influenced by bilateral gaze distractors.

The flanker task in Experiment 1 was mainly used to investigate the mutual disturbance effect between a target and distractors. However, both gaze and arrow cues carry directional information involving spatial coding. For example, previous researchers have shown that gaze and arrow cues could induce attention shifts so that they could lead to Simon effect (Ansorge, 2003; Miles & Proctor, 2012; Zorzi et al., 2003). It means that spatial information may also be processed even when a gaze cue and an arrow cue is presented as a target or distractor. Then, how is their processing affected by irrelevant spatial conflict? Will they show similar processing patterns under the influence of a spatial distractor? If spatial coding plays an important role in processing gaze and arrow cues, responses to them may be impeded when the direction they point to is incongruent with the location at which they are presented. Experiment 2 addressed these issues by manipulating the congruency between the directions of gaze and arrow cues and their relative positions in a spatial compatibility task.

## Experiment 1a

### Methods

#### Participants

Twenty-one undergraduate students (9 men and 12 women; aged 18–25 years; *M* = 21.32 years, standard deviation [*SD*] = 1.43) with normal or corrected-to-normal vision participated in the present study. The study was approved by the local ethics committee, and informed consent was obtained from participants prior to the commencement of the experiment.

##### Apparatus and stimuli

A central cross (diameter 0.3°) was used as a fixation point. The stimuli were a pair of black arrows pointing leftward or rightward (0.45° in height and 0.9° in width) inside a black rectangle (1.14° in height and 2.2° in width) and a pair of eyes gazing leftward or rightward (0.45° in height and 0.9° in width) inside an identical rectangle (Bonato et al., 2009). All experimental materials were drawn in black lines. Three arrays of stimuli were presented simultaneously in every trial, including a central target and distractors on both sides. Arrows and gaze cues had an equal possibility to be the central target or bilateral distractors, and they could point in the same or opposite direction. Participants were required to respond rapidly and accurately to only the direction of the central target and ignore the bilateral distractors.

Stimulus arrays were displayed on a white background on a Founder 170 CRT monitor with a resolution of 1,024 × 768 pixels and a refresh rate of 85 Hz. The participants were seated 70 cm away from the computer screen, and they responded by pressing one of two designated buttons on a standard keyboard. We controlled stimulus presentations and collected behavioural data using E-prime 1.1 software (Psychology Software Tools, Inc., Sharpsburg, PA).

##### Design and procedure

The experiment used a 2 (target: arrow and gaze) × 2 (type congruency between target and distractor in flanker task: type-congruent vs. type-incongruent) × 2 (direction congruency between the target and distractor: direction-congruent vs. direction-incongruent) within-subject design.

**Figure 1.**
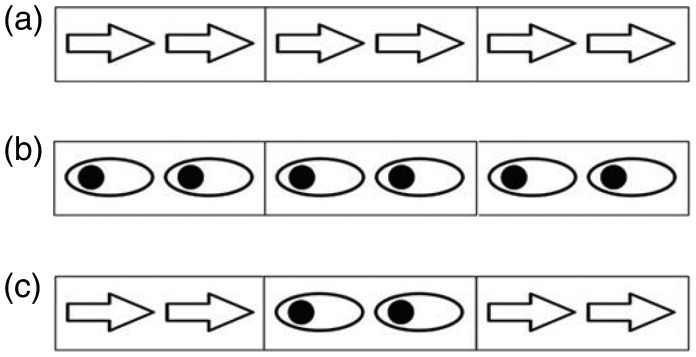
Samples of stimuli presented in three conditions in Experiment 1a. (a) Arrows in direction-congruent (direction-con) and type-congruent (type-con) trials, (b) gaze in direction-con and type-con trials, and (c) gaze in direction-incongruent (direction-incon) and type-incongruent (type-incon) trials.

At the start of each trial, a black fixation cross appeared for 500 ms at the centre of the screen. Then, arrays of stimuli in the flanker task were presented for 1,500 ms. Three arrays of stimuli were presented simultaneously in every trial, including a central target and bilateral distractors. Samples of presented stimuli have been depicted in Figure 1. Arrow and gaze cues had an equal possibility to be the central target or distractors on both sides and could point in the same or opposite direction with the same probability. Participants responded rapidly and accurately to the direction of the central target by pressing ‘N’ for the left, using the index finger of their right hand, and ‘M’ for the right, using the middle finger of their right hand, ignoring the bilateral distractors. The next trial began after a blank screen was shown for 1,000 ms. All conditions were randomized across the experimental session. The formal experiment was conducted after 16 practice trials. There were a total of 640 experimental trials, containing 4 blocks of 160 trials each.

#### Results

##### Accuracy

The mean accuracies were analysed with a 2 (central target: arrow and gaze) ×2 (type congruency: type-con and type-incon) ×2 (direction congruency: direction-con and direction-incon) repeated measures analysis of variance (ANOVA). There were significant main effects of central target, *F*(1, 20) = 12.79, *p* < .01, $\eta_{\text{p}}^{2}$ = 0.39, power (1 − *β*) = 0.93; type congruency, *F*(1, 20) = 5.54, *p* < .05, $\eta_{\text{p}}^{2}$ = 0.21, power (1 − *β*) > 0.99; and direction congruency, *F*(1, 20) = 15.23, *p* < .01, $\eta_{\text{p}}^{2}$ = 0.43, power (1 − *β*) = 0.96, with higher accuracy (ACC) in trials with arrow targets (98.9%) than that in trials with gaze targets (98.3%), in trials with the type-con condition (98.8%) than in those with the type-incon condition (98.3%), and in trials with the direction-con condition (99.0%) than in those with the direction-incon condition (98.1%). Further, the three-way interaction among central target, direction congruency and type congruency was significant, *F*(1, 20) = 4.85, *p* < .05, $\eta_{\text{p}}^{2}$ = 0.19, power (1 − *β*) = 0.55. The two-way interactions between central target and direction congruency, *F*(1, 20) = 7.45, *p* < .05, $\eta_{\text{p}}^{2}$ = 0.27, power (1 − *β*) = 0.73; central target and type congruency, *F*(1, 20) = 6.71, *p* < .05, $\eta_{\text{p}}^{2}$ = 0.25, power (1 − *β*) = 0.69; direction congruency and type congruency, *F*(1, 20) = 9.43, *p* < .01, $\eta_{\text{p}}^{2}$ = 0.32, power (1 − *β*) = 0.83, were also proved significant. Simple effect tests revealed that there were more errors in direction-incon trials (ACC = 96%) than that in direction-con trials (ACC = 99%) when responding to gaze flanked by arrows, *F*(1, 20) = 12.88, *p* < .01, $\eta_{\text{p}}^{2}$ = 0.39, power (1 − *β*) > 0.99. Bonferroni corrections were made for the tests. Conditions with smaller reaction times corresponded to those with higher ACC. Thus, there was no indication of a speed–ACC trade-off (see Table 1).

**Table 1.** Mean Response Time (ms), Accuracy (%) and Standard Deviation to the Two Types of Direction Congruency (Direction-Con and Direction-Incon) and Two Types of Type Congruency (Type-Con and Type-Incon) in Different Central Target Conditions (Arrow and Gaze) in Experiment 1a.

Central target, type congruency and direction congruency	*M* (*SD*)	Accuracy (*SD*)
Arrow		
Type-con		
Direction-con	484 (43)	0.98 (0.01)
Direction-incon	507 (42)	0.98 (0.01)
Type-incon		
Direction-con	488 (42)	0.99 (0.01)
Direction-incon	493 (40)	0.98 (0.02)
Gaze		
Type-con		
Direction-con	503 (46)	0.99 (0.02)
Direction-incon	524 (45)	0.98 (0.01)
Type-incon		
Direction-con	506 (48)	0.99 (0.01)
Direction-incon	555 (45)	0.96 (0.01)

##### Response times

Only correct responses were included in the analysis, and trials with response times (RTs) exceeding three *SD*s from the overall mean for each participant (<3% of trials) were excluded from the analysis (see Table 1). The mean RTs were examined using a 2 (central target: arrow and gaze)×2 (type congruency: type-con and type-incon) × 2 (direction congruency: direction-con and direction-incon) repeated measures ANOVA. This analysis confirmed the main effects of central target, *F*(1, 20) = 242.44, *p* < .001, $\eta_{\text{p}}^{2}$ = 0.64, power (1 − *β*)>0.99; type congruency, *F*(1, 20) = 20.98, *p* < .001, $\eta_{\text{p}}^{2}$ = 0.53, power (1 − *β*)>0.99; and direction congruency, *F*(1, 20) = 122.58, *p* < .001, $\eta_{\text{p}}^{2}$ = 0.52, power (1 − *β*)>0.99, with faster RTs in trials with arrow targets (493.23 ms) than in those with gaze targets (522.63 ms), in trials with the type-con condition (504.5 ms) than in those with the type-incon condition (510.98 ms), and in trials with the direction-con condition (495.73 ms) than in those with the direction-incon condition (519.97 ms), indicating the presence of flanker effects (RT_direction-incon_ − RT_direction-con_).

The three-way interaction among central target, type congruency and direction congruency was significant, *F*(1, 20)  =  40.98, *p* < .001, $\eta_{\text{p}}^{2}$ = 0.36, power (1 − *β*) > 0.99. Further, significant two-way interactions were found between central target and type congruency, *F*(1, 20) = 54.41, *p* < .001, $\eta_{\text{p}}^{2}$ = 0.43, power (1 − *β*) > 0.99; central target and direction congruency, *F*(1, 20)  =  57.13, *p* < .001, $\eta_{\text{p}}^{2}$ = 0.66, power (1 − *β*) > 0.99. Simple effect tests revealed that, in arrow trials, a significantly slower RTs were found in direction-incon than in direction-con trials in the type-con condition: *MD* = 23 ms, *F* (1, 20)  =  51.71, *p* < .001, $\eta_{\text{p}}^{2}$ = 0.72, power (1 − *β*) > 0.99, but not in the type-incon condition (*MD* = 5 ms), indicating that these have a flanker effect in the type-con condition but not in the type-incon condition. Whereas, in gaze trials, significantly slower RTs were found in the direction-incon than direction-con trials not only in the type-con condition: *MD* = 21 ms, *F* (1, 20) = 32.98, *p* < .001, $\eta_{\text{p}}^{2}$ = 0.62, power (1 − *β*) > 0.99, but also in the type-incon condition: *MD* = 49 ms, *F* (1, 20) = 1.09, *p* < .001, $\eta_{\text{p}}^{2}$ = 0.84, power (1 − *β*) > 0.99. Bonferroni corrections were made for the tests.

**Figure 2.**
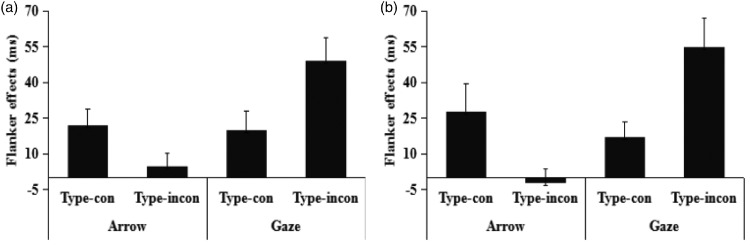
Flanker effect (RT_direction-incon_ − RT_direction-con_) of each condition in Experiments 1a (a) and 1b (b). Error bars represent standard errors.

We further compared the magnitude of the flanker effect for gaze or arrow cues separately and found that the flanker effect of gaze cues was larger in the type-incon condition (49 ms) than in the type-con condition (21 ms), *t*(20) = 4.87, *p* < .001, Cohen’s *d*: 1.06, power (1 − *β*) > 0.99, while the flanker effect of arrow cues was smaller in the type-incon condition (5 ms) than in the type-con condition (23 ms), *t* (20) = 4.68, *p* < .001, Cohen’s *d*: 1.02, power (1 − *β*) > 0.99 (see Figure 2(a)).

## Experiment 1b

Results from Experiment 1a showed that although gaze was largely affected by bilateral arrow distractors, arrow as well as gaze cues produced similar flanker effects when the bilateral distractors were congeneric, which may indicate the presence of reflexive attention induced by irrelevant arrow and gaze cues. However, in Experiment 1a, participants were required to press the left or right key to respond to the directions of a gaze or arrow cue pointing left or right. Thus, stimuli and response in Experiment 1a shared a common spatial property, which may produce spatial stimuli–response compatibility effects (Eimer, 1995; Fitts & Seeger, 1953). Previous evidence has shown that the gaze-induced cueing effect was influenced by the Simon effect, as evidenced by the stronger cueing effect for valid gaze cues when the response key and target location were on the same side (McKee, Christie, & Klein, 2007). Further, a study conducted by Zorzi et al. (2003) directly investigated whether irrelevant gaze direction information automatically produced the Simon effect when responding to the colour of gaze ‘irises’. They found that although gaze cues were presented in the centre of the screen with its direction irrelevant to the discrimination task, faster response time was observed when the location of the response key was in line with the gaze direction than when it was not. This was called the ‘gaze direction Simon effect’ (see Ansorge, 2003). Arrows, as symbolic direction cues, might also produce the Simon effect, even when presented in the centre of the screen (Masaki, Takasawa, & Yamazaki, 2000). Therefore, to control the interference from stimuli–response compatibility effects in Experiment 1b, we used a subset of 21 new participants (five men and 16 women; 18-25 years; M = 21.55 years, SD = 1.54) in the same task as Experiment 1a, using ‘2’ and ‘5’ on the keypad as response keys, which were different from the direction of the targets.

### Results

#### Accuracy

The mean accuracies were analysed with a 2 (central target: arrow and gaze) × 2 (type congruency: type-con vs. type-incon) × 2 (direction congruency: direction-con vs. direction-incon) repeated measures ANOVA. There were significant main effects of central target, *F*(1, 20)  =  8.62, *p* < .01, $\eta_{\text{p}}^{2}$ = 0.30, power (1 − *β*)  =  0.80; type congruency, *F*(1, 20)  =  9.49, *p* < .01, $\eta_{\text{p}}^{2}$ = 0.32, power (1 − *β*)  =  0.83; and direction congruency, *F*(1, 20) = 14.47, *p* < .001, $\eta_{\text{p}}^{2}$ = 0.42, power (1 − *β*) = 0.95, with higher ACC in trials with arrow targets (99.1%) than that in trials with gaze targets (97.7%), in trials with the type-con condition (98.7%) than in those with the type-incon condition (98.0%), and in trials with the direction-con condition (99.0%) than in those with the direction-incon condition (97.8%). The three-way interaction among central target, direction congruency and type congruency was significant, *F*(1, 20) = 11.72, *p* < .01, $\eta_{\text{p}}^{2}$ = 0.37, power (1 − *β*) = 0.9. Further, significant two-way interactions were found between central target and direction congruency, *F*(1, 20) = 4.44, *p* < .05, $\eta_{\text{p}}^{2}$ = 0.18, power (1 − *β*) = 0.52; central target and type congruency, *F*(1, 20) = 3.69, *p* = .06, $\eta_{\text{p}}^{2}$ = 0.15, power (1 − *β*) = 0.44; direction congruency and type congruency, *F*(1, 20) = 8.11, *p* < .05, $\eta_{\text{p}}^{2}$ = 0.28, power (1 − *β*) = 0.77. Simple effect tests revealed that higher ACC in the direction-con trials (99%) than in the direction-incon trials (95%) was found when responding to a gaze cue flanked by arrows, *F*(1, 20) = 14.05, *p* < .01, $\eta_{\text{p}}^{2}$ = 0.41, power (1 − *β*) > 0.99. Bonferroni corrections were made for the tests. Conditions with smaller reaction times corresponded to those with higher ACC. Thus, there was no indication of a speed–ACC trade-off (see Table 2).

**Table 2.** Mean Response Time (ms), Accuracy (%) and Standard Deviation to the Two Types of Direction Congruency (Direction-Con and Direction-Incon) and Two Types of Type Congruency (Type-Con and Type-Incon) in Different Central Target Conditions (Arrow and Gaze) in Experiment 1b.

Central target, type congruency and direction congruency	*M* (*SD*)	Accuracy (*SD*)
Arrow		
Type-con		
Direction-con	445 (54)	0.99 (0.01)
Direction-incon	474 (56)	0.98 (0.01)
Type-incon		
Direction-con	446 (53)	0.99 (0.01)
Direction-incon	444 (51)	0.98 (0.01)
Gaze		
Type-con		
Direction-con	465 (58)	0.98 (0.02)
Direction-incon	482 (52)	0.98 (0.02)
Type-incon		
Direction-con	466 (57)	0.99 (0.01)
Direction-incon	522 (57)	0.95 (0.05)

#### Response times

Only correct responses were included in the analysis, and trials with RTs exceeding three *SD*s from the overall mean for each participant (<3% of trials) were excluded from the analysis (see Table 2). The mean RTs were examined using a 2 (central target: arrow and gaze) × 2 (type congruency: type-con vs. type-incon) × 2 (direction congruency: direction-con vs. direction-incon) repeated measures ANOVA. This analysis revealed the significant main effects of central target, *F*(1, 20) = 120.51, *p* < .001, $\eta_{\text{p}}^{2}$ = 0.85, power (1 − *β*) > 0.99, and direction congruency, *F*(1, 20) = 170.31, *p* < .001, $\eta_{\text{p}}^{2}$ = 0.89, power (1 − *β*) > 0.99, with faster RTs in trials with arrow targets (452.65 ms) than that in trials with gaze targets (484.20 ms), and in trials with the direction-con condition (456.03 ms) than in those with the direction-incon condition (480.82 ms).

The three-way interaction among central target, type congruency and direction congruency, *F*(1, 20) = 59.12, *p* < .001, $\eta_{\text{p}}^{2}$ = 0.74, power (1 − *β*) > 0.99, was significant. Significant two-way interactions were also found between central target and type congruency, *F*(1, 20) = 60.30, *p* < .001, $\eta_{\text{p}}^{2}$ = 0.75, power (1 − *β*) > 0.99; central target and direction congruency, *F*(1, 20) = 29.92, *p* < .001, $\eta_{\text{p}}^{2}$ = 0.56, power (1 − *β*) > 0.99. Simple effect tests revealed that, in arrow trials, a significantly slower RT in direction-incon than direction-con trials was found in the type-con condition: *MD* = 28 ms, *F* (1, 20) = 30.65, *p* < .001, $\eta_{\text{p}}^{2}$ = 0.61, power (1 − *β*) > 0.99, but not in the type-incon condition (*MD* = −2 ms), indicating that these cues have a flanker effect in the type-con but not in the type-incon condition. Whereas, in gaze trials, significantly slower RTs in the direction-incon than in the direction-con trials was observed not only in the type-con condition: *MD* = 17 ms, *F* (1, 20) = 35.85, *p* < .001, $\eta_{\text{p}}^{2}$ = 0.64, power (1 − *β*) > 0.99, but also in the type-incon condition: *MD* = 55 ms, *F* (1, 20) = 1.08, *p* < .001, $\eta_{\text{p}}^{2}$ = 0.84, power (1 − *β*) > 0.99. Bonferroni corrections were made for the tests.

By comparing the magnitude of the flanker effect for gaze or arrow cues separately, we found that the flanker effect of gaze cues was larger in the type-incon condition (55 ms) than that in the type-con condition (17 ms), *t*(20) = 6.69, *p* < .001, Cohen’s *d*: 1.45, power (1 − *β*) > 0.99, while the flanker effect of arrow cues was smaller in the type-incon condition (−2 ms) than that in the type-con condition (28 ms), *t* (20) = 5.06, *p* < .001, Cohen’s *d*: 1.10, power (1 − *β*) > 0.99 (see Figure 2(b)).

#### Discussion

Through Experiment 1a, we investigated the flanker effect of gaze and arrow cues when they competed with each other. Similar results were found in Experiment 1b, ruling out the possible interference on the results by the congruency between the target’s direction and response key’s location. Therefore, we could draw two main conclusions from Experiment 1. First, we found that gaze and arrow cues triggered the same flanker effect in the type-congruency condition, which revealed that, although participants were instructed to ignore the bilaterally presented gaze and arrow distractors, the distractors were still processed unintentionally and both types produced interference effects on target processing. This finding demonstrated that both gaze and arrow cues might trigger reflexive attentional orientations such that they disturbed the response to the target even in a task-irrelevant state (Bonato et al., 2009). Second, an asymmetric flanker effect for gaze and arrow cues was found in the type-incon condition: The flanker effect for a gaze cue increased when the distractors were arrows, while the flanker effect of an arrow cue decreased or even disappeared when the distractors were gaze cues. Specifically, the processing of an arrow in the flanker task was less disturbed by gaze distractors as compared to the effect of arrow distractors on the processing of the gaze target.

This finding might suggest a more strongly reflexive attention triggered by arrow cues as compared to that triggered by gaze cues. Moreover, considering the shorter RTs for responding to arrow direction than to gaze direction in Experiments 1a and 1b, another explanation might also be noticed. It was possible that both gaze and arrow cues triggered reflexive attentional processes, but the perceptual difference between gaze and arrow cues resulted in the asymmetric conflict effect when they competed with each other in the flanker task (Horstmann et al., 2006; Nummenmaa & Hietanen, 2009). Specifically, as compared with the gaze condition, arrow direction might be more salient and easy to distinguish (Nummenmaa & Hietanen, 2009). When manipulating arrow and gaze cues simultaneously in a trial, the processing of the central arrow was more rapid than that of the bilateral gaze distractors, but the processing of a central gaze cue was relatively more time-consuming than that of the bilateral arrow distractors was. Therefore, responding to the direction of a central arrow was less influenced by bilateral gaze distractors, but responding to the direction of gaze was less interfered by bilateral arrow distractors. In addition, the process of focusing attention is considered necessary for processing gaze cues while orienting attention, which explains why the gaze-induced reflexive attention was weak when they were presented as bilateral distractors (Ansorge, 2003; Marotta et al., 2013).

## Experiment 2a

Experiment 2 aimed to investigate how the processing of gaze and arrow directions is affected by irrelevant spatial conflict, and whether they show similar processing patterns under the influence of a spatial distractor. Using a spatial compatibility task, a gaze cue or an arrow cue was presented as a target in each trial, and the compatibility between the direction of the gaze or arrow cue and their relative spatial locations were manipulated. For example, a spatial conflict situation may be produced when an arrow pointing to the left is presented to the right of the central fixation. If spatial coding plays a similar role in the processing of the direction of gaze and arrow cues, responses to them may be impeded when the direction they point to is incongruent with the location at which they are presented.

### Methods

#### Participants

Thirty-six undergraduate students (seven men and 29 women; 18-25 years; M = 21.33 years, SD = 1.47) with normal or corrected-to-normal vision participated in the present study. The study was approved by the local ethics committee, and informed consent was obtained from participants prior to the commencement of the experiment.

##### Apparatus and stimuli

The experiment was controlled by E-Prime software using a PC with a 17-in. CRT monitor. The participant was seated in front of the screen. The distance between the screen and participants’ eyes was 70 cm. A central cross was used as a fixation point. The stimuli were a pair of black arrows pointing leftward or rightward inside a rectangle and a pair of eyes gazing leftward or rightward inside an identical rectangle. All experimental materials were drawn using black lines. Arrays of stimuli had an equal possibility of appearing in the horizontal right, left, top and bottom positions, at a 4.5° visual angle from the fixation cross.

##### Design and procedure

**Figure 3.**
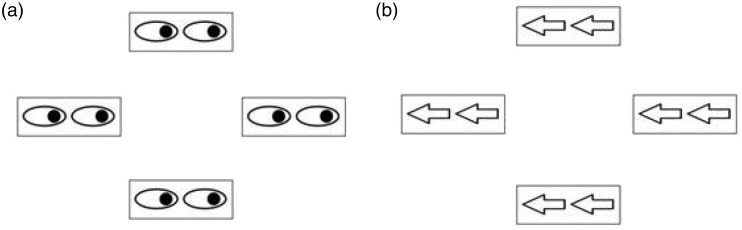
Samples of stimuli presented in Experiment 2a. A gaze or arrow cue was presented only at one of the four locations for each trial. (a) Gaze-corresponding trials: A gaze cue directing right was presented on the right side of the screen; gaze-non-corresponding trials: A gaze cue directing right was presented on the left side of the screen; gaze-unrelated trials: A gaze cue directing right was presented above or below the centre fixation on the screen. (b) Arrow-corresponding trials: An arrow cue directing right was presented on the right side of the screen; arrow-non-corresponding trials: An arrow cue directing right was presented on the left side of the screen; arrow-unrelated trials: An arrow cue directing right was presented above or below the centre fixation on the screen.

A trial started with the presentation of a fixation cross. After fixation, a pair of gaze or arrow cues directed right or left appeared for 1,500 ms in one of the four locations, each with an equal possibility of appearance: left of, right of, above and below the fixation. Samples of stimuli presented have been depicted in Figure 3. The directions of the gaze and arrow cues might differ from, be the same as, or be unrelated to the relative spatial locations of the gaze or arrow targets. Participants responded accurately and rapidly only to the direction of the presented gaze or arrows by pressing ‘N’ for left with the index finger of their right hand and ‘M’ for the right with the middle finger of the right hand, ignoring the relative spatial location. The next trial began after a blank screen was presented for 1,000 ms. All conditions were randomized across the experimental session. The formal experiment was performed after 18 practice trials. There were a total of 480 experimental trials containing 4 blocks of 120 trials each.

The experiment followed a 2 (target: arrow and gaze) × 3 (location-direction correspondence: corresponding, non-corresponding and unrelated) within-subject design. All the conditions were manipulated factorially within the participants. Under both corresponding and non-corresponding conditions, a gaze or arrow cue directing left or right was presented on the right or left side of the screen. The direction of the gaze or arrow cue was consistent with its location in corresponding trials and was opposite from its location in non-corresponding trials. In unrelated trials, a gaze or arrow cue that directed left or right was presented above or below the centre fixation on the screen.

#### Results

##### Accuracy

The mean accuracies were analysed with a 2 (target: arrow and gaze) × 3 (location-direction correspondence: corresponding, non-corresponding and unrelated) repeated measures ANOVA. A significant main effect was found for location-direction correspondence, *F*(2, 70) = 4.16, *p* < .01, $\eta_{\text{p}}^{2}$ = 0.19, power (1 − *β*) = 0.86, with higher ACC in unrelated (97%) and corresponding trials (97%) than in non-corresponding trials (95%). No other significant effects were found. Conditions with smaller reaction times corresponded to those with higher ACC. Thus, there was no indication of a speed–ACC trade-off (see Table 3).

**Table 3.** Mean Response Time (ms), Accuracy (%) and Standard Deviation to the Three Types of Location-Direction Correspondence (Corresponding, Non-Corresponding and Unrelated) in Different Target Conditions (Arrow and Gaze) in Experiment 2a.

Target and location-direction correspondence	*M* (*SD*)	Accuracy (*SD*)
Arrow		
Corresponding	506 (45)	0.97 (0.03)
Non-corresponding	520 (49)	0.94 (0.05)
Unrelated	531 (51)	0.96 (0.03)
Gaze		
Corresponding	532 (49)	0.96 (0.03)
Non-corresponding	528 (54)	0.95 (0.04)
Unrelated	531 (52)	0.97 (0.03)

##### Response times

Only correct responses were included in the analysis, and trials with RTs exceeding three *SD*s from the overall mean for each participant (<3% of trials) were excluded from the analysis (see Table 3). The mean RTs were obtained using a 2 (target: arrow and gaze) × 3 (location-direction correspondence: corresponding, non-corresponding and unrelated) repeated measures ANOVA. The main effect of the target was significant, *F*(1, 35) = 9.507, *p* < .01, $\eta_{\text{p}}^{2}$ = 0.45, power (1 − *β*) = 0.85. The response to arrow targets (519 ms) was faster than that to gaze targets (531 ms). A significant main effect was also observed for the location-direction correspondence, *F*(2, 70) = 7.07, *p* < .01, $\eta_{\text{p}}^{2}$ = 0.29, power (1 − *β*) = 0.75. The response time in the unrelated condition (531 ms) was longer than that in the corresponding (519 ms) and non-corresponding conditions (524 ms). Furthermore, the interaction between target type and location-direction correspondence also reached significance, *F*(2, 70) = 11.67, *p* < .001, $\eta_{\text{p}}^{2}$ = 0.41, power (1 − *β*) > 0.99. Bonferroni-corrected pairwise tests showed that RTs in the gaze trials were significantly greater than those in the arrow trials for the corresponding condition (532 vs. 506 ms), *t*(35) = 5.11, *p* < .001, Cohen’s *d*: 0.82, power (1 − *β*) > 0.99, and marginally significant for those in the non-corresponding condition (528 vs. 520 ms), *t*(35) = 1.87, *p* = .07, Cohen’s *d*: 0.30, power (1 − *β*) = 0.54. However, no difference was found between gaze and arrow trials in the neutral condition (531 vs. 531 ms), *t*(35) = 0.30, *p* > .9, power (1 − *β*) = 0.05.

Moreover, we calculated the location-conflict effect in gaze and arrow trials, respectively, by subtracting RTs for the corresponding condition from those for the uncorresponding condition. Then, we compared the effect under the gaze and arrow conditions and found that the location-conflict effect was larger for arrow trials (14 ms) than it was for gaze trials (−4 ms), *t*(35) = 3.23, *p* < .01, Cohen’s *d*: 0.52, power (1 − *β*) = 0.95.

## Experiment 2b

Experiment 2a showed that the attentional mechanisms of gaze and arrow cues differed when conflicting spatial information was presented. However, in Experiment 2a, responding to gaze and arrow cues using the left or right response keys ‘N’ and ‘M’, respectively, was expected to lead to at least two kinds of spatial Simon effects that may affect the participants’ responses to stimuli’s directions. One is the classical spatial Simon effect triggered by the congruency between stimuli’s and response key’s spatial location (Zorzi & Umiltà, 1995). The other is the stimuli-direction Simon effect induced by the congruency between the stimuli’s direction and the response key’s location (Zorzi et al., 2003). Thus, in order to rule out those two kinds of Simon effects that may also explain the main results obtained in Experiment 2a, we utilized a subset of 21 new participants (4 men and 17 women; 18–25 years; *M* = 21.62 years, *SD* = 1.52) in the same task as Experiment 2a in Experiment 2b and used the up/down response keys of ‘2’ and ‘5’ on the keypad.

### Results

#### Accuracy

The mean accuracies were analysed with a 2 (target: arrow and gaze) × 3 (location-direction correspondence: corresponding, non-corresponding and unrelated) repeated measures ANOVA. A significant main effect was found for location-direction correspondence, *F*(2, 40) = 12.70, *p* < .001, $\eta_{\text{p}}^{2}$ = 0.38, power (1 − *β*) > 0.99, with higher ACC in unrelated (98%) and corresponding trials (97%) than in non-corresponding trials (95%). No other significant effects were found. Conditions with smaller reaction times corresponded to those with higher ACC. Thus, there was no indication of a speed–ACC trade-off (see Table 4).

**Table 4.** Mean Response Time (ms), Accuracy (%) and Standard Deviation to the Three Types of Location-Direction Correspondence (Corresponding, Non-Corresponding and Unrelated) in Different Target Conditions (Arrow, Gaze) in Experiment 2b.

Target and location-direction correspondence	*M* (*SD*)	Accuracy (*SD*)
Arrow		
Corresponding	547 (55)	0.97 (0.03)
Non-corresponding	560 (60)	0.96 (0.04)
Unrelated	568 (57)	0.97 (0.03)
Gaze		
Corresponding	571 (60)	0.97 (0.03)
Non-corresponding	567 (55)	0.95 (0.04)
Unrelated	565 (54)	0.98 (0.01)

#### Response times

Only correct responses were included in the analysis, and trials with RTs exceeding three *SD*s from the overall mean for each participant (<3% of trials) were excluded from the analysis (see Table 4). The mean RTs were examined using a 2 (target: arrow and gaze) × 3 (location-direction correspondence: corresponding, non-corresponding and unrelated) repeated measures ANOVA. The main effect of the target was significant, *F*(1, 20) = 7.23, *p* < .05, $\eta_{\text{p}}^{2}$ = 0.26, power (1 − *β*) = 0.82. The response to arrow targets (558 ms) was faster than that to gaze targets (567 ms). Furthermore, the interaction between target type and location-direction correspondence was significant, *F*(2, 40) = 11.67, *p* < .001, $\eta_{\text{p}}^{2}$ = 0.42, power (1 − *β*) = 0.98. Bonferroni-corrected pairwise *t* tests showed that RTs in the gaze trials were significantly greater than those in the arrow trials for the corresponding (571 vs. 547 ms), *t*(20) = 4.22, *p* < .001, Cohen’s *d*: 0.92, power (1 − *β*) > 0.99, and non-corresponding conditions (567 vs. 560 ms), *t*(20) = 2.11, *p* < .05, Cohen’s *d*: 0.46, power (1 − *β*) = 0.68. However, no difference was found between gaze and arrow trials in the unrelated condition (568 vs. 565 ms), *t*(20) = 0.70, *p* > .49, power (1 − *β*) = 0.16.

Moreover, we also calculated the location-conflict effect in gaze and arrow trials, respectively, by subtracting the RTs for the corresponding condition from those for the uncorresponding condition. Then, we compared the effect in the gaze and arrow conditions and found that the location-conflict effect was larger for arrow trials (12 ms) than for gaze trials (−3 ms), *t*(20) = 2.61, *p* < .05, Cohen’s *d*: 0.56, power (1 − *β*) = 0.8.

#### Discussion

We can obtain two conclusions from the results of Experiment 2. First, consistent with the result of Experiment 1, participants responded faster to arrows than to gaze, indicating that arrow cues can be responded rapidly. Second, a significant difference in the response time in the location-direction corresponding condition and non-corresponding condition was found only when responding to the arrow cue but not to the gaze cue, showing that the processing of gaze was independent of spatial coding, but the processing of arrows was susceptible to irrelevant spatial information. Results from Experiment 2b ruled out the interference from the response key’s location. Less impact on the processing of gaze cues by irrelevant spatial information, as compared with the processing of arrow cues, may demonstrate a divergent or specialized mechanism for gaze processing such that it is more difficult to be interfered by conflicting spatial information. These results were supported by previous studies showing that the processing of gaze direction was independent of stimulus spatial coding (Zorzi et al., 2003). Similar results were also found by Ansorge (2003), which revealed that the Simon effect of the observe-relative screen position of faces was absent if the processing of the observed gaze direction was required.

## General Discussion

The present study directly explores how gaze and arrow are processed when facing competing distractors by presenting them as targets or distractors in flanker tasks and in spatial compatibility tasks. In Experiment 1, the processing of gaze and arrow targets was equally disturbed by congeneric distractors and produced the same flanker effect, showing similar reflexive attentional effects of gaze and arrow distractors. However, an asymmetric conflict effect was found when gaze and arrow competed with each other in the flanker task: Arrow distractors greatly interfered with the attentional processing of gaze, while the processing of arrow direction was immune to conflict from gaze distractors. Experiment 2 revealed that spatial interference effects were only found for arrows but not for gaze cues when they were presented as targets, indicating attention triggered by gaze was less affected by conflicting spatial location information. Different effects on gaze and arrows by different types of conflicts reflect two relatively distinct specific modes of the attentional process. Specifically, processing to arrow cues is less influenced by bilateral gaze cues but is affected by irrelevant spatial information, while processing to a gaze cue is greatly disturbed by bilateral arrows but is unaffected by irrelevant spatial information.

For the conflict task in the congruent-type trials (arrow target with arrow distractors or gaze target with gaze distractors), the bilateral distractors showed similar interference on target processing regardless of type, as reflected by the equal flanker effect. This outcome reveals that, even when presented as irrelevant distractors, gaze and arrow cues can be processed reflexively, and they can disturb the processing of the target. This part of the conclusion seems consistent with the results found in the previous literature, which showed that both arrow and gaze cues can produce reflexive attentional orientation as directional cues even when they are irrelevant to the current task (Nummenmaa & Hietanen, 2009; Tipples, 2008). When participants respond to the direction of a central gaze or arrow, they are strongly motivated to inhibit the spatial orientation information provided by adjacent gaze or arrow distractors to ensure the efficiency of target detection. If the flankers behave voluntarily, they are easy to inhibit and produce no effect on target processing. However, we found that the response to gaze is slower when the bilateral gaze shows a conflicting direction, demonstrating that gaze distractors indeed induce spatial information processing, and they are difficult to inhibit. Attentional processing of an arrow target shows the same conflict effect when competing with adjacent arrows pointing in an opposite direction. Therefore, even when gaze and arrows are considered as distractors and not directional cues predicting the possible location of an incoming target, they can reflexively elicit spatial direction information that cannot be ignored (Galfano et al., 2012).

For the conflict task in incongruent-type trials (arrow target with gaze distractors or gaze target with arrow distractors), asymmetric conflict effects were found, as reflected by a larger flanker effect for gaze than for arrow targets. Arrows seem to win the attentional priority in the competition for attentional resources due to their rapid processing as targets and inefficient inhibition as distractors, which means that different attentional mechanisms may be involved in the processing of gaze and arrow cues (Zhou & Liu, 2013). It seems that arrow cues trigger a more reflexive spatial orientation of visual attention than gaze cues do in the flanker task. Moreover, to explain these results, we turn to our finding of faster RTs for arrow than for gaze; this may suggest that perceptual processing speeds for gaze and arrows are not equal when both cues simultaneously appeared. When responding to a gaze cue flanked by two arrows, arrow distractors might be processed before the central gaze, which results in greater interference with the processing of gaze direction. The study by Horstmann et al. (2006) identified that perceptual differences between the stimuli can account for the flanker-effect asymmetry.

Faster overall response time for arrow targets than that for gaze targets in both Experiments 1 and 2 also provided evidence for the above-mentioned explanation. This result was also supported by some studies reporting overall faster RTs for arrows than for gaze cues (Nummenmaa & Hietanen, 2009). Driver et al. (1999) reasoned that the encoding of gaze direction could be more time-consuming than the detection of a peripheral cue with an abrupt onset is. Arrows can be rapidly processed, probably because the arrow is a well-learned symbol that conveys strong spatial information that is reinforced every day, for instance, by means of road signs (Galfano et al., 2012). There are also some differences between the presentation of gaze as cues and their presentation as distractors. Gaze cues are characterized by a stronger biological significance than an arrow, since humans have developed a reflexive attentional shift in response to an averted gaze. However, when gaze cues are presented as targets, the biological significance of gaze may decrease. Gaze can be considered relatively more complex stimuli that need more time to be processed as compared to arrows. Further, observers are not sensitive to the eye directions of faces that are presented outside the focus of attention, which may also explain why the processing of an arrow target was less interfered by bilateral gaze cues (Ansorge, 2003).

Experiment 2 further compared the spatial location conflict effect of gaze and arrow cues and found the different effects on gaze cues and arrow cues by irrelevant spatial information. Responding to the direction-location corresponding arrows (arrows pointing left and appearing on the left of the screen) was more efficient than that to the direction-location conflict arrows (arrows pointing left but appearing on the right of the screen). Yet the response to gaze was independent of spatial locations, which might reveal that gaze, as a social direction cue, was less affected by other factors in the environment, and it was processed in a more reflexive and unique manner. Similar conclusions were obtained by some investigators. Using a typical Simon task, they found that focusing of attention on the eyes prior to response selection presumably rendered the faces’ lateral positions neutral such that the coding of the gaze direction was independent of stimulus spatial coding. This finding verified the hypothesis that gaze direction was coded by a specialized mechanism (Ansorge, 2003).

Altogether, different effects on gaze cues and arrow cues by different types of conflicts may reflect two divergent attentional processes to gaze and arrow cues (Zhao, Uono, Yoshimura, & Toichi, 2014). Previous studies have also found different processing patterns for gaze and arrows as directional cues (Hayward & Ristic, 2015; Zhao, Li, Uono, Yoshimura, & Toichi, 2017). For instance, Langdon and Smith (2005) found that gaze cues triggered facilitated and inhibitory cueing effects, whereas arrow cues triggered facilitated and inhibition-less priming effects, which supported the view that, unlike arrows, gaze cues were unique symbolic directional cues. Furthermore, a study by Marotta et al. (2012) suggested that attention was non-specifically directed to nearby objects when a non-informative arrow was used as a cue, whereas it was selectively directed to a specific cued location when non-informative eye gaze was used. Importantly, results from the current study directly verified the hypothesis that gaze cues represented a divergent attentional process to arrows by presenting gaze and arrows as targets or distractors (Brignani, Guzzon, Marzi, & Miniussi, 2009; Zhao, Uono, Yoshimura, & Toichi, 2015).

In summary, using a flanker task and spatial compatibility task, the present study explored the attentional mechanisms of gaze and arrows when they are presented as targets or distractors and when they compete with irrelevant distractors. Different types of conflicts affect the attentional processing of gaze and arrow targets differently. Gaze and arrow targets are equally disturbed by congeneric distractors but are divergently affected by heterogenetic distractors, which indicate that, although gaze and arrow cues share the characteristic of reflexive attention, the processing of arrow direction may be more rapid. Interestingly, processing to gaze cues is greatly disturbed by arrows but is unaffected by irrelevant spatial information, reflecting the divergent attentional processing to a gaze cue compared to an arrow cue when facing spatial conflict. This is in line with the findings of previous studies that eye direction and arrow direction trigger similar reflexive shifts in spatial attention, but that they also have some difference in triggering reflexive attention (Feng & Zhang, 2014; Feng et al., 2011; Langdon & Smith, 2005; Ristic et al., 2007). However, previous studies focused on the comparison between mechanisms of gaze and arrow cues separately. Therefore, our research extends the existing literature and further probes the attentional mechanisms and conflict effects by presenting them as target or distractors in a trial, which provides more evidence for the similarities and differences between the processing mechanisms of gaze and arrow cues.

Some limitations of this study should be acknowledged. First, to identify the similarities between gaze and arrow cues, the gaze materials used in the present study are schematic pictures with less ecological validity. Second, the present study explores only two types of directional signs; therefore, other biological cues, including digit and symbol cues, and social cues, such as face orientation and finger pointing, require further investigation. Third, we did not run a preliminary power analyses to determine the sample sizes before the experiments. However, by complementally running a preliminary power analyses, we found that in order to detect medium-size effects (size *f* = 0.25) at the desired significance levels of *α* = *β* = 0.5, a sample of *N* = 16 is adequate to provide 80% power to detect key findings of Experiment 1, and a sample of *N* = 19 is adequate to provide 80% power to detect key findings of Experiment 2. In the current study, the number of participants in Experiment 1 (*N* = 21) and Experiment 2 (*N* = 36) is more than that. Also, we have run a post hoc analysis to determine the power of the current studies following anonymous reviewers’ suggestion. As we reported in our results, statistical power (1 – *β*) of critical results ranged between 0.83 and 0.99 in Experiments 1 and 2, which indicated that the sample sizes we used are adequate to support the results.
